# Group cognitive behavior therapy for Japanese patients with social anxiety disorder: preliminary outcomes and their predictors

**DOI:** 10.1186/1471-244X-7-69

**Published:** 2007-12-10

**Authors:** Junwen Chen, Yumi Nakano, Tetsuji Ietzugu, Sei Ogawa, Tadashi Funayama, Norio Watanabe, Yumiko Noda, Toshi A Furukawa

**Affiliations:** 1 Department of Psychiatry and Cognitive-Behavioral Medicine, Nagoya City University Graduate School of Medical Sciences, Nagoya, Japan; 2 Department of Psychology, Faculty of Human Relations, Tokai Gakuin University, Gifu, Japan; 3 Matsukage Hospital, Nagoya, Japan

## Abstract

**Background:**

A number of studies have provided strong evidence for the use of cognitive behavior therapy (CBT) in the treatment of social anxiety disorder (SAD). However, all of the previous reports were from Europe and North America and it is unknown whether Western psychological therapies are effective for SAD in non-Western cultures. The present pilot study aimed to evaluate CBT program for SAD which was originally developed for Western patients, among Japanese patients.

**Methods:**

Fifty-seven outpatients who participated in group CBT for SAD were evaluated using eight self-reported and one clinician-administered questionnaires to measure various aspects of SAD symptomatology at the beginning and at the end of the program. Pre- and post-treatment scores were compared and the magnitude of treatment effect was quantified as well based once on the intention-to-treat (ITT) and once among the completers only. We also examined baseline predictors of the CBT outcomes.

**Results:**

Seven patients (12%) did not complete the program. For the ITT sample, the percentage of reduction was 20% to 30% and the pre to post treatment effect sizes ranged from 0.37 to 1.01. Among the completers, the respective figures were 20% to 33% and 0.41 to 1.19. We found no significant pretreatment predictor of the outcomes.

**Conclusion:**

Group CBT for SAD is acceptable and can bring about a similar degree of symptom reduction among Japanese patients with SAD as among Western patients.

## Background

Social anxiety disorder, with life time prevalence over 10% [1], is said to be the most prevalent anxiety disorder in some Western countries. Curiously enough, the reported prevalence of SAD is much lower in East Asian countries and East African countries, where it or related disorders have been believed to be culture-bound [2]. In order to understand this important disorder, Clark and Wells [3], and Rapee and Heimberg [4] have developed cognitive models that focus on its maintenance mechanisms. In the treatment of SAD, a number of studies have provided strong evidence for the use of cognitive behavior therapy (CBT). Five meta-analyses have found large effect sizes ranging from 0.80 to 1.07 for the reduction of social anxiety symptoms [5-8]. Moreover, some researchers also demonstrated that group administered CBT is cost-effective [6] and is associated with lower relapse rates during follow-up period than phenelzine [9]. Recent researches based on the model of Clark and Wells [3] have shown superior outcomes and the effect sizes of the CBT group were substantially larger than those reported by other research groups, suggesting that these new methods are superior to other CBT programs [10,11].

However, all of these previous reports were mostly from Europe and North America. Cultural factors are especially relevant [12,2]. For example, Taijin-Kyofu-Sho (TKS), which is listed in the appendix to DSM-IV, is said to be a culture-bound syndrome unique to East Asia. Moreover, CBT models and treatment components for SAD were mostly developed in Western cultures with theoretical orientations typically constrained by Western conceptualizations of anxiety. Translations and modifications are needed when a treatment program is introduced to Japan to make it appropriate to the Japanese culture. To date, it is still unknown whether Western psychological therapies are effective and acceptable for patients with SAD and related disorders in non-Western cultures. In addition, most of the reported studies have been conducted in research settings and thus do not reflect usual patient samples in clinical settings. Whether the treatment can be delivered with the same effectiveness in a more 'naturalistic' medical routine remains unproven. Taken together, it is necessary to investigate whether CBT can achieve comparable treatment outcomes for SAD in non-Western cultures.

The present pilot study aims to report the preliminary outcomes of the CBT program for SAD at Department of Psychiatry at Nagoya City University Hospital and to examine the baseline predictors of the outcomes of Japanese patients with SAD receiving CBT.

Nagoya City University Hospital is located in Nagoya City, Japan, and is affiliated with the Nagoya City University School of Medicine. It has both inpatient and outpatient sections. In the outpatient section, CBT program for SAD as well as other anxiety and mood disorders are carried out as a part of routine clinical work.

## Methods

### Subjects

Fifty seven consecutive patients with SAD were recruited into the outpatient group-based CBT program at the Department of Psychiatry, Nagoya City University Hospital, Japan between July 2003 and January 2007. Some patients were referred from mental health professionals and others sought treatment for social anxiety disorder on their own.

All patients were diagnosed with SAD as the primary disorder according to the DSM-IV criteria, as assessed by the Structured Clinical Interview for DSM-IV [13]. In addition, all patients fulfilled the following criteria: (a) absence of a history of psychosis or bipolar disorder, and of current substance use disorder; and (b) no previous CBT treatments, nor any other additional psychosocial therapies during the treatment, and (c) absence of Cluster B personality disorder. With the exception of criterion (c), current Axis I major depressive disorder, other current anxiety disorders and Axis II personality disorders were not a reason for exclusion if the patients' symptoms abated enough to allow them to attend the group CBT sessions regularly.

The patients provided their written informed consent after full explanation of the objectives and procedures of the present study. The study's protocol was approved by the Ethics Committee of Nagoya City University Graduate School of Medical Sciences.

### Treatments

Initially we started the program with 12 two-hour sessions, adopting the model developed by Gavin Andrews *et al.*[14] in Sydney, Australia. As we modified and improved the program according to Clark & Wells' model [3], the program was extended to 12 or more sessions depending on each group's needs. After this modification, the average number of sessions per group was 15 (range: 11 to 20).

The program is based on Clark & Wells' model, which focuses on the maintenance of SAD. The main components include 1) psychoeducation regarding the nature of anxiety and symptoms of SAD, 2) deriving an individualized version of the cognitive-behavioral model of SAD using patient's own thoughts, images, anxiety symptoms, safety behaviors and strategies of attention, 3) experiment to drop safety behaviors and self-focused attention, 4) attention training to shift focus of attention externally to the task at hand or the social situation, 5) video feedback of role-plays of moderately anxiety-provoking situations to modify distorted self-imagery, 6) behavioral experiments to test the patients' catastrophic predictions during planned exposure to the situations in within-session role-plays and in vivo homework assignments, and 7) cognitive restructuring for identifying and modifying dysfunctional assumptions especially during behavioral experiments. Homework was assigned after every session.

The patients were treated in groups of 3 or 4 led by two therapists (one principal therapist and one co-therapist). This format was adopted because we found it was too difficult to provide each one out of four or more patients with the individual attention that is needed for the detailed practices of CBT techniques. Most of the patients appeared to be comfortable with this format. Eight therapists (five psychiatrists and three doctoral-level clinical psychologists) with more than three years of clinical practice and experience in treatment of anxiety disorders conducted the treatment program guided by a therapist manual in Japanese. During the treatment, therapists had group discussion once a month to check adherence to the program and to plan for future sessions. However, the quality of treatment delivery was not rated though audio or video recordings.

During the CBT treatment, co-administration of antidepressants and benzodiazepines was allowed because there is no evidence to contraindicate their concurrent use [15,16], and also because symptoms of other comorbid anxiety and/or depressive disorders had to be controlled with antidepressants.

### Assessment

Patients were assessed with a structured diagnostic interview as well as an extensive questionnaire battery. At baseline, before treatment commencement, the principal therapist in charge of the CBT group administered the mood and anxiety disorder sections of the Structured Clinical Interview for DSM-IV [13], in order to ascertain SAD diagnosis and any mood and anxiety comorbidities. In addition, eight self-reported and one clinician-administered questionnaires to measure various aspects of SAD symptomatology and functional impairment, broader psychopathology in general, and premorbid personality were administered at the same time. The same instruments, except for the personality inventory, were repeated at the end of the program.

#### Social Phobia Scale and Social Interaction Anxiety (SPS/SIAS)

The SPS/SIAS [17] is a 20-item self-report questionnaire respectively. The SPS was designed to measure the fear of being observed whereas the SIAS provides a measure of fear of social interaction. Each scale has twenty items rated on a 4-point scale, from 0 (not at all characteristic or true of me) to 4 (extremely characteristic or true of me), with scores for each scale ranging from 0 to 80. Excellent internal consistency reliability and sufficient discriminant, predictive and concurrent validity have been demonstrated for both of the Japanese versions [18].

#### Fear Questionnaire – social phobia subscale (FQ-sp)

The FQ-sp is a 5-item self-reported instrument for measuring the fear-motivated avoidance of being observed, performing, being criticized, and talking to authorities [19]. Items are rated on a 9-point Liker-type scale, from 0 (would not avoid it) to 8 (always avoid it). Good test-retest reliability and factor validity have been demonstrated [19,20].

#### The Fear of Negative Evaluation Scale (FNE) and Brief Fear of Negative Evaluation Scale (BFNE)

The FNE is a 30-item questionnaire developed to measure fear of negative evaluation in social situations [21]. Items are rated as true or false, with the total score ranging from 0 to 30. Good reliability and validity of the Japanese version have been reported [22]. However, the newer brief version of the FNE contains 12 items with a 5-point Likert-type format (1 = "not at all characteristic of me"; 5 = "extremely characteristic of me") has been found to be more sensitive to treatment-related change [23]. We therefore switched to BFNE after several groups. As the BFNE was not given to patients from the beginning, calculations can only be made for a smaller sample of n = 37 (FNE) and n = 26 (BFNE).

#### The Liebowitz Social Anxiety Scale (LSAS)

The LSAS is the most frequently used clinician-administered instrument for the assessment of social phobia. It is a 24-item scale that provides separate scores for fear (0–3 indicate none, mild, moderate, and severe, respectively) and avoidance (0–3 indicate never, occasionally, often, and usually, respectively) of social interaction and performance situations, thus providing scores of four subscales, Fear of Performance, Avoidance of Performance, Fear of Social Interaction, and Avoidance of Social Interaction. The Cronbach's alpha reliability estimate of the Japanese version is 0.95 for total items and 0.80–0.91 for subscales. In addition, sufficient validity data have also been provided [24].

#### Symptom Checklist-90-Revised (SCL-90-R)

The SCL-90-R is a 90-item questionnaire widely used for the assessment of general psychopathology [25]. It yields scores for nine primary symptom dimension of somatization, obsessive-compulsive, interpersonal sensitivity, depression, anxiety, hostility, phobic anxiety, paranoid ideation, and psychosis. The reliability and validity of the Japanese version have been reported [26].

#### Work, Home Management, Social and Private Leisure Activities Scale (WHLS)

The WHLS is a self-report scale for measuring functional impairments in the areas of work, home management, social and private activities [19]. The patients' answers are graded between 0 (not at all impaired) to 8 (very severely impaired). Satisfactory reliability and construct validity have been reported [27].

#### NEO Five Factor Index (NEO-FFI)

This is a 60-item self-reported questionnaire designed to measure the five major personality dimensions of neuroticism, extraversion, conscientiousness, openness and agreeableness [28]. The Japanese version has been tested in a general population and satisfactory internal consistency (coefficient alpha) is provided as well as validity [29].

### Statistical analyses

First, treatment completers were compared with patients dropping out during treatment using unpaired *t *tests for continuous variables or chi-squared tests for categorical variables.

Second, the outcomes of the CBT program for the Japanese patients with SAD were quantified in the following ways. Our primary outcome was the total LSAS score, and percentages of patients with symptomatic response and remission were calculated. Following Bandelow *et al.*[30], treatment responder status is defined as 31% or greater reduction in the LSAS and remission as a score of 36 or less on the LSAS.

Furthermore, to examine the outcomes of the CBT program across various aspects of the disorder, pre- and post-treatment scores were compared for each SAD symptomatological scale (SPS, SIAS, FQ-sp, FNE, BFNE, LSAS) as well as the functional scale (WHLS) using paired *t *tests. The magnitude of treatment effect was quantified in two ways by way of the percentage of reduction ((pre – post-treatment)/pretreatment scores × 100) as well as the effect size (*(M*_*pretest*_-*M*_*posttest*_)/*SD*_*pretest*_). According to Cohen [31], effect sizes are categorized as follows: small (0.20–0.49), medium (0.50–0.79), and large effects (0.80 and above).

All statistical analyses for these treatment outcomes were conducted twice, once based on the intention-to-treat (ITT) principle whereby all the dropouts were considered to be non-responders and their last available observations were carried forward, and once among the completers only. Treatment completers were defined as participants who had attended at least 80 percent of the treatment sessions and returned post treatment questionnaires. The ITT analyses present the more conservative estimates of treatment effectiveness.

Third, in order to elucidate the baseline predictors of the treatment outcomes, multiple regression analyses were conducted with the post-treatment LSAS score as a dependent variable and the baseline demographic and clinical variables as independent variables, while controlling for the baseline LSAS scores. These analyses were performed among the completers only.

All the statistical tests were two-tailed, and an alpha value of less than 0.05 was considered statistically significant. All the data were examined using SPSS 15.0 for Windows [SPSS Inc., 2007].

## Results

### Demographic and Diagnostic Characteristics of the Patients

Table 1 summarizes the demographic and clinical characteristics of patients. All participants met the principal diagnostic criteria for DSM-IV SAD. Additional Axis I diagnoses for the patients included other anxiety disorders (14%), dysthymia (19.3%), and major depressive disorder (38.6%).

**Table 1:** Demographic and clinical characteristics of the patients

Age (years), Mean (SD)	30.4 (10.3)
Age of onset (years), Mean (SD)	17.3 (6.2)
Duration of SAD (years), Mean (SD)	13.3 (10.4)
Gender	
Male, n (%)	28 (49.1)
Female, n (%)	29 (50.9)
Marital status	
Single, n (%)	40 (70.2)
Married, n (%)	16 (28.1)
Divorced, n (%)	1 (1.7)
Education	
University, n (%)	24 (42.9)
College, n (%)	11 (19.6)
High school, n (%)	21 (37.5)
Subtype	
Generalized, n (%)	46 (80.7)
Comorbidity	
With comorbidity, n (%)	34 (59.6)
Without comorbidity, n (%)	23 (40.4)
Medication	
Benzodiazepine (BZ), n (%)	14 (24.6)
Antidepressant (AD), n (%)	28 (49.1)
Both BZ & AD use, n (%)	33 (57.9)

### Comparison of Treatment Completers and Dropouts

Of the 57 patients who agreed to undergo treatment, 7 (12.3%) dropped out before program completion. The following reasons were given for dropping out: (a) the therapy seemed too difficult to pursue (n = 1), (b) organizational difficulties due to changes in life styles (e.g. move back home and too far to come, started working) (n = 2), (c) there was improvement in symptoms due to the patients' own views (n = 1), (d) increased anxiety (n = 3). A comparison of demographic and pretreatment variables in the questionnaires revealed no significant differences between dropouts and treatment completers (all *p *values > 0.10).

### Percentage of Patients with Symptomatic Response and Remission

For the ITT sample, the mean number of sessions attended were 14.0 (SD= 4.3), while 15.3 (SD = 2.5) for completers (n = 50). Nineteen (33.3%) patients were judged to be responders and 10 (18.5%) of the patients met criterion for remission at post treatment on the ITT basis including dropouts. The respective figures were 38.0% and 21.3% among the completers.

### Changes in Symptoms and Functions through the Treatment

Table 2 shows the means and standard deviations of all the measures for all the participants (intention-to-treat) and completers at pre- and post-treatment.

**Table 2:** Intention-to-treat and completers mean symptom scores and standard deviations at pre- and post-treatment

	**Intention-to-treat (n = 57)**		**Completers (n = 50)**	
	Pre	Post	Pre	Post

Social Phobia Scale	36.2 (15.4)	24.7 (15.2) ***	36.7 (15.4)	24.0 (15.3) ***
Social Interaction Anxiety Scale	54.8 (15.0)	43.0 (16.0) ***	54.9 (14.8)	41.8 (15.6) ***
Fear of Negative Evaluation^1^	24.6 (5.1)	22.1 (6.5) **	24.3 (5.1)	20.8 (6.5) **
Brief Fear of Negative Evaluation^2^	33.2 (10.7)	28.1 (9.6) **	33.4 (10.6)	27.6 (9.3) ***
FQ-sp^a^	24.0 (6.2)	17.8 (7.8) ***	24.2 (6.2)	16.9 (8.1) ***
WHLS^b^	14.0 (6.1)	10.4 (6.3) ***	13.9 (6.1)	9.4 (6.3) ***
				
LSAS (total)^c^	76.2 (25.6)	59.6 (27.3) ***	77.2 (26.1)	58.5 (27.8) ***
Fear of performance	20.8 (5.8)	16.3 (6.9) ***	20.9 (6.1)	15.9 (7.2) ***
Avoidance of performance	17.3 (8.2)	11.5 (7.9) ***	17.8 (8.3)	11.1 (8.0) ***
Fear of social interaction	20.0 (6.9)	17.5 (7.2) **	20.1 (6.8)	17.3 (7.2) **
Avoidance of social interaction	18.1 (7.9)	14.3 (8.5) ***	18.4 (7.8)	14.2 (8.4) ***
				
NEO Five Factor Index				
Neuroticism	31.0 (9.0)	-	31.6 (9.2)	-
Extroversion	21.8 (8.0)	-	21.6 (8.1)	-
Conscientiousness	24.7 (7.7)	-	24.6 (8.0)	-
Openness	30.5 (6.7)	-	29.0 (5.3)	-
Agreeableness	24.2 (7.9)	-	30.3 (6.8)	-
				
Symptom Checklist-90 Revised				
Somatization	0.7 (0.6)	0.5 (0.6) n.s.	0.7 (0.6)	0.5 (0.5) **
Obsessive-compulsive	1.7 (0.9)	1.4 (0.9) **	1.7 (0.9)	1.3 (0.8) **
Interpersonal sensitivity	2.1 (0.8)	1.6 (0.9) ***	2.1 (0.8)	1.4 (0.9) ***
Depression	1.6 (0.8)	1.2 (0.9) ***	1.6 (0.8)	1.1 (0.8) ***
Anxiety	1.5 (0.8)	1.2 (0.9) **	1.4 (0.8)	1.1 (0.9) **
Hostility	0.8 (0.6)	0.6 (0.6) **	0.8 (0.6)	0.6 (0.6) **
Phobic anxiety	1.1 (0.8)	0.7 (0.7) ***	1.1 (0.8)	0.7 (0.6) ***
Paranoid ideation	1.1 (0.8)	0.8 (0.9) **	1.1 (0.7)	0.8 (0.8) **
Psychoticism	0.8 (0.5)	0.6 (0.6) ***	0.9 (0.5)	0.6 (0.6) ***

FQ-sp^a^: Fear Questionnaire Social Phobia Subscale

WHLS^b^: Work, Home and Leisure Activities Scale

LSAS (total)^c^: Liebowitz Social Anxiety Scale (total)

Fear of Negative Evaluation^1^: Number for ITT = 37, Number for Completers' analysis = 27

Brief Fear of Negative Evaluation^2^: Number for ITT = 26, Number for Completers' analysis = 23

(*** *p *< 0.001, **p < 0.01)

Examination of change in symptom measures (the LSAS, SPS, SIAS, FQ-sp, FNE, BFNE) and functional impairment scale (the WHLS) between pre and post-treatment revealed significant improvements, not only for the ITT sample but also for the completers (all *p *values < 0.01).

Next, the percentage of reduction and effect size in each symptom measures was calculated and the results are presented in Table 3.

**Table 3:** Percentage of reduction and effect size for symptom scales (Intention-to-treat and completers)

	***Intention-to-treat***		***Completers***	
	***% of reduction***	**Effect size**	***% of reduction***	**Effect size**
		
Social Phobia Scale	30	0.75	33	0.83
Social Interaction Anxiety Scale	21	0.79	23	0.89
Fear of Negative Evaluation	9	0.49	12	0.68
Brief Fear of Negative Evaluation	12	0.48	20	0.54
FQ-sp^a^	25	1.01	30	1.19
				
Liebowitz Social Anxiety Scale (total)	20	0.65	22	0.71
Fear of performance	21	0.78	23	0.83
Avoidance of performance	26	0.71	31	0.80
Fear of social interaction	10	0.37	11	0.41
Avoidance of social interaction	20	0.47	23	0.53
				
WHLS^b^	20	0.59	36	0.75

FQ-sp^a^: Fear Questionnaire Social Phobia Subscale

WHLS^b^: Work, Home and Leisure Activities Scale

For the intention-to-treat sample, the percentage of reduction was 20% to 30% for most of the symptom measures and 20% for WHLS. However, a lower improvement in FNE (9%), BFNE (12%) and fear of social interaction subscale of LSAS (10%) were seen for all the participants.

Furthermore, based on the analyses of ITT sample, effect size for the social phobic measures (SPS, SIAS, FNE, BFNE, FQ-sp, and LSAS total and subscale scores) at post assessment ranged from 0.37 to 1.01 and was 0.59 on WHLS (see Table 3). According to Cohen's classification [31], FQ-sp displayed largest effects whereas the other social phobic measures showed moderate effects at best. Moreover, FNE, BFNE, Fear of social Interaction subscale and Avoidance of Social Interaction subscale of LSAS were associated with a small effect sizes at post treatment.

Similar to the ITT sample, completer analyses indicated a 20% to 33% percentage of reduction for most of the symptom measures and 36% for the WHLS. A lower percentage of reduction was also seen in FNE (12%) and fear of social interaction subscale of LSAS (11%).

For treatment completers, effect sizes at post assessment ranged from 0.41 to 1.19 on the social phobic measures which indicated a greater change than the ITT sample. Among the outcome variables, not only FQ-sp, but also SIAS, SPS, Fear of performance subscale and Avoidance of performance subscale of LSAS displayed large effect sizes. In addition, effect size of WHLS was 0.75.

### Predictors of Treatment Response

Multiple regression analyses, using the baseline LSAS score and each demographic variable (age, gender, marital status, educational status, age of onset, duration of disorder, subtypes, presence of comorbid, use of anxiolytic medication, or use of antidepressant medication, number of treatment sessions) as independent variables and the post treatment LSAS score as a dependent valuable, were conducted. None of the variables were significant predictors of post treatment LSAS score.

The last analysis addressed the predictive relationship between NEO and SCL pretreatment scores and post treatment LSAS score. We carried out two separate, multiple linear regression analyses with the five subscales of NEO and the nine subscales of SCL-90-R as the respective explanatory variables, and the post treatment LSAS score as the dependent variable, while controlling the pretreatment LSAS score. However, neither NEO nor SCL-90-R appeared as a predictor in these analyses.

## Discussion

Firstly, the present study demonstrated that a CBT program for SAD, originally developed in Western countries, is feasible and effective in a Japanese clinical setting. To date, cultural impact is said to be relevant to the prevalence, presentation, and treatment of SAD [2]. In Japan, for example, TKS is said to be a Japanese conceptualization of SAD. Although TKS and SAD are both characterized by excessive fear and avoidance of social interaction and performance, the concerns in TKS center on offending or embarrassing others [32]. Despite a better understanding of the variation in symptom presentation of some of the trans-cultural variants of SAD, such as TKS, little is known about the usefulness of Western psychological therapies for it. In addition, various methodological explanations can be offered to explain cross-cultural differences or the absence of expected findings. For example, not only the use of assessment instruments but the treatment components which were developed in English and translated into Japanese may affect the outcome of CBT program. Therefore, it is necessary to investigate whether an equal treatment outcome can be achieved when transporting the Western developed CBT to Japan. Irrespective of cultural background, our results suggested that the CBT program appears to be equally acceptable to Japanese patients with SAD as to Western patients, because our dropout rate (12.3%) is generally lower than those reported in Western studies of group CBT (see Table 4) and comparable to some individual CBT programs [33,34].

**Table 4:** Comparison of effect sizes by group CBT

	Completers analyses									ITT					
Study	Heimberg (1998)			Stangier (2003)			Present study			Mörtberg (2007)			Present study		
Group	12 weeks			15 weeks			16 weeks			3 weeks			16 weeks		
N	36			26			57			35			57		
Drop out (%)	22.2			15.4			12.3			25.7			12.3		

	Pre	Post	ES	Pre	Post	ES	Pre	Post	ES	Pre	Post	ES	Pre	Post	ES
					
LSAS total	59.0	46.2	0.97				77.2	58.5	0.71	68.1	52.5	0.75	76.2	59.6	0.65
SD	13.2	8.9					26.1	27.8		20.9	19.4		25.6	27.3	
Social fear	15.1	12.0	0.44				20.1	17.3	0.41				20.0	17.5	0.37
SD	7.0	4.9					6.8	7.2					6.9	7.2	
Social avoidance	13.1	9.9	0.42				18.4	14.2	0.53				18.1	14.3	0.47
SD	7.7	4.8					7.8	8.5					7.9	8.5	
Performance fear	17.0	13.4	0.65				20.9	15.9	0.83				20.8	16.3	0.78
SD	5.6	3.8					6.1	7.2					5.8	6.9	
Performance avoidance	13.8	10.9	0.47				17.2	11.1	0.80				17.3	11.5	0.71
SD	6.0	4.3					8.3	8.0					8.2	7.9	
FQ-sp^a^	19.0	14.7	0.60				24.2	16.9	1.19	20.7	15.5	0.78	24.0	17.8	1.01
SD	7.2	4.0					6.2	8.1		6.7	7.1		6.2	7.8	
SPS	27.3	25.8	0.09	33.6	25.5	0.53	36.7	24.0	0.83	36.5	27.7	0.64	36.2	24.7	0.75
SD	17.0	10.0		15.3	15.3		15.4	15.3		13.8	11.5		15.4	15.2	
SIAS	41.8	39.4	0.13	45.8	38.4	0.53	54.9	41.8	0.89	44.5	36.7	0.48	54.8	43.0	0.79
SD	18.4	13.2		14.0	12.4		14.8	15.6		16.1	14.7		15.0	16.0	
FNE	22.3	21.4	0.12				24.3	20.8	0.68	22.5	21.5	0.15	24.6	22.1	0.49
SD	7.6	4.3					5.2	6.5		6.5	7.2		5.1	6.5	

FQ-sp^a^: Fear Questionnaire Social Phobia Subscale

In terms of effectiveness, our CBT program also compares favorably with Western reports. For example, our group CBT program was able to reduce most of the symptom measures by 20 to 30%, figures comparable to those reported in Western settings [33-35]. Several previous meta-analyses of CBT for SAD have derived effect sizes based on within-group change from pre to post treatment between 0.51 to 1.06 for completers [5,7,8]. Within-group effect sizes in our program were largely consistent with these figures (see Table 3). However, effect sizes reported in the previous studies were calculated by various methods from various outcome measures. For a direct comparison with studies of group CBT conducted recently using similar SAD symptomatological scales, we calculated the effect sizes based on these scales using the formula M_pretest_-M_posttest_/SD_pretest_. As shown in Table 4, our pre to post treatment effect sizes were superior to those of the previous studies of group CBT with regard to FQ-sp, SPS, SIAS, and subscales of LSAS (Fear of performance, Avoidance of performance, Avoidance of social interaction). However, two recent RCTs demonstrated superiority of individual format CBT over group format [33,34]. Our program was carried out in groups of only 3 to 4 patients in order to allow as much individual attention as possible while retaining the advantage of group settings (e.g. role plays and learning through peers) and therefore can be thought to be more individualized than most reported group CBT programs (6–8 patients per group, and fixed sessions). However, the existence of a similar advantage of strictly individual format among Japanese patients has yet to be investigated.

Moderate effect sizes were demonstrated for WHLS, indicating that the impact of SAD on patients' daily lives had reduced. However, there are very few studies that used WHLS as an outcome measure, so that a direct comparison was not possible here. Similarly, a direct comparison of symptomatic remission with prior studies was also not possible because of imprecise description in many of the studies.

In addition to the outcomes described previously, we conducted a preliminary examination on whether there are any differences between patients with SAD and TKS. Six patients (10.5%) met the criteria of TKS and there were no statistical and substantial differences between patients with SAD and TKS in terms of LSAS, SPS or SIAS both before and after the treatment (For TKS, the means (SD) of LSAS, SPS and SIAS were 75.5 (43.5), 35.3 (17.4), 45.8 (19.1) at baseline, and 61.3 (49.9), 33.8 (21.8) and 43.0 (24.0) at end of treatment).

Secondly, identifying possible predictors of treatment response in the treatment completers showed that none of the pretreatment variables were significant predictors of scores of LSAS at post treatment. A number of studies have examined the role of particular variables in predicting response to treatment and their influence on overall therapeutic outcome but the results were inconsistent and inconclusive [36]. The fact that no predicting effect emerged indicates that pretreatment variables may have little to offer for the prediction of treatment outcome. However, there are some other variables we did not directly measure in our study. For example, expectancy for improvement is said to be related to outcome [36]. Future studies should also focus not only on pretreatment variables but also processes during the treatment, such as homework compliance and the client-therapist relationship which was suggested by Scholing *et al.*[37].

The present study has several limitations. First, this is a preliminary study which was conducted in a Japanese routine clinical setting. As we modified and improved the treatment program based on the latest theoretical model, it may raise a problem of treatment consistency. A further study should examine whether there are differences in treatment outcome due to different CBT models. Moreover, lack of supervision may also have decreased treatment consistency and adherence to treatment. In Japan, there are not enough professional therapists for CBT available in routine medical settings. Regular supervision from experts in cognitive therapy is necessary and will further strengthen the training system.

Second, it must be pointed out that in the present study the principle therapist conducted the diagnostic assessments before and after the treatment, which may cause detection bias as well as performance bias. Independent blind assessor ratings would have strengthened our conclusions. Moreover, repeated *t*-test for comparing pre-treatment with post-treatment scores may have increased the risk of Type I error. Thus, some non-important differences may have been falsely found to be statistically significant. However, the magnitude of treatment effect was quantified by effect size as well as the percentage of reduction and the results are clearly demonstrated.

Third, the inclusion of subjects using psychotropic medication, although increasing the generalizability of the study, also confounds the treatment results because ethically we could not prohibit medication changes while subjects participated in the study. However, previous researches have failed to find a significant advantage for combined pharmacotherapy and CBT [10,15,16]. Rosser *et al.*[38] investigated the impact of preexisting antidepressant use on the outcome of group CBT for SAD in a naturalistic routine clinical setting and also failed to find a significant difference between combined treatments and CBT alone. In our study, none of the pretreatment variables were significant predictors of scores of LSAS at post treatment suggested that medication may have little impact on the treatment outcome. Furthermore, for those 25 (50%) patients who completed the treatment and who had already been on medication upon study participation, we examined the medication regimen at the end of treatment. Twelve (25%) of them were stabilized on the regimen throughout the treatment, while 8 (16%) had their medication reduced, and 3 (6%) had it increased. No data was available for two of the patients because they had their prescription filled elsewhere. In other words, over 90% of the subjects had their medication status unchanged or decreased through the study.

Nevertheless, considering that antidepressant medication and benzodiazepines are effective for the treatment of SAD [15], the current study did not answer questions as to whether the treatment outcome is due to the contribution of medication or CBT alone. Future controlled trials need to explore the issue whether pre-existing medication has a significant impact on treatment outcome and also to address the question of whether treatment effects are maintained over the longer term to an equivalent extent for CBT alone, pharmacotherapy alone, or a combined approach.

Fourth, our sample size was relatively small and thus power was limited, especially with regard to predictive variables. Moreover, lack of control group data limits the generalizability of the results and lack of follow-up data limits the ability of the study to comment on longer term outcomes. Future studies including larger sample and control group should provide more insight into the CBT treatment in Japanese routine medical settings.

Despite the limitations, this pilot study provided an independent CBT treatment within a routine psychiatric service in a different cultural setting from the Western countries. It reported a preliminary treatment outcome in Japan in comparison with those from Western countries. Although there is still room for improvement, our results suggested a general replication of CBT for SAD in Japan.

## Conclusion

CBT program for SAD, originally developed in Western countries, is acceptable to Japanese patients in a routine clinical setting. Within-group changes from pre to post treatment obtained by group CBT program are comparable to those reported in Western settings.

## Competing interests

The author(s) declare that they have no competing interests.

## Authors' contributions

JC was the primary investigator; JC, YNakano, TI, SO, TF, NW, YNoda, TAF performed the clinical investigation (diagnosis, treatment and assessment); TAF participated in the design of the study and supervised the overall conduct of the study. All authors read and approved the final manuscript.

## Pre-publication history

The pre-publication history for this paper can be accessed here:


